# The Popular Conception of the Specialist

**Published:** 1912-03-30

**Authors:** 


The Hospital

The Workers' Newspaper of Administrative Medicine and Institutional Life, Administration, National Insurance and Health.

No. 1342, Vol. LI. SATURDAY, MARCH 30, 1912. PRICE ONE PENNY.

### THE POPULAR CONCEPTION OF THE SPECIALIST.

During the last few months, more perhaps than ever before, the members of the medical profession, collectively and individually, have been very prominently "in the public eye." The Insurance Act, with its attendant talk of a nationalised medical service, and the light that a discussion of certain of its clauses has thrown upon the mysteries of club and hospital practice, have been largely instrumental in thus throwing the limelight on the doctor, and more especially upon the consultant. For it is the consultant who after all, so far as the public is concerned, represents the profession. So long as a medical man lives in Harley Street—for Wimpole, Weymouth, and Queen Anne Streets, and Devonshire and Portland Places, and even Cavendish Square, are hardly considered the holiest of holies by those who write about the doctor in the lay Press or discuss him at the clubs—he has an infallible claim to be considered a consultant, and, being a consultant, to be a specialist, a little lower than the angels but many, many steps above the general practitioner no matter in what quarter of the Empire. That is the popular qualification for a consultant, or rather, the popular conception of a qualification. Curiously enough, the people who have framed this conception would be the last persons in the world to give to Robert Street in Liverpool, or Karlstrasse in Berlin, the same pre-eminence as the abode of the "Levites" as they ascribe to Harley Street. The conception of a consultant, so far as the average member of the public is concerned, is not that of a man who has by hard work, honest endeavour, and patient research made his way to the top of the tree; it is merely that of a man who has advertised himself, first by taking rooms in Harley Street—a method which is now so much overdone that it hardly ever suffices to bring in a quarter's rent—and secondly by hitting the public fancy with some fad or some coquetting with that publicity which runs dangerously close to the unethical crime condoned only when committed in the first manner. We see this idea of the doctor's personality and quality illustrated in the fairly vivid sketches drawn of their medical characters by popular novelists. Sir Conan Doyle's picture of the consultant-club doctor in "The Stark Munro Letters" is so obviously a caricature that it will not pass for a moment, while the expert knowledge of the author saves him from erring in those very points that are mysteries to the public. Mr. E. F. Benson has given us a very carefully drawn delineation of what he considers a specialist in Dr. Compton. Perhaps it is not quite the popular conception, for Mr. Benson is a faithful student of psychology, and even the profession, taking an unprejudiced view of its own portrait, will have to admit that some of the lines are very true. Dr. Compton, who specialises in nerves, is apparently attached to no hospital but has a very large practice: his personal portrait is drawn in a sentence: "he had a face like a rather good-looking monkey, shrewd, plain for a human being, and almost painfully intelligent"—a description in which the jam so carefully conceals the pill that it will pass almost for a compliment. Mr. Benson's specialist is a gentleman who discusses his patients with outsiders, dabbles in spiritualism, and who in half a dozen other ways shows the initiated that he could not possibly have been a "Levite."

But he answers to the popular conception and is glibly accepted by thousands of readers just as the public gratefully accept the unutterable trash of which "a Harley Street specialist" in the recurring interview in the halfpenny Press unbosoms himself on such questions as alcoholism, degeneration, eugenics, sleeplessness, vegetarianism, the suffrage question, the decline of the birth-rate, a new anæsthetic, or a new method of serum treatment for hay fever. The fact remains that the public finds it wholly impossible to gauge the medical profession by the standards which we apply ourselves. It measures all by the same rule and applies to everyone the one invariable test—residence in Harley Street. Here and there when a provincial specialist of established reputation receives the favour of public notice he has to be apologised for, and it has to be laboriously explained how and why he is famous, just as a well-known newspaper recently found it expedient to tell its readers who

Professor Ehrlich was by comparing him with the late Sir William Broadbent! The cure for this misconception of the specialist is only to be found in the education of both the public and the profession. The former will have to be taught that so long as there is no one portal of entrance to the profession there must of necessity be various ranks of "Levites," and that the only criterion of a man's standing is his work: the latter will have to lay aside the armour of indifference, and expose shams and counterfeits. A man has no more right to pose as a specialist if he has not specialised than he has to betray the confidence of his patients or patent his prescriptions.

